# *Burkholderia pseudomallei*, the causative agent of melioidosis, is rare but ecologically established and widely dispersed in the environment in Puerto Rico

**DOI:** 10.1371/journal.pntd.0007727

**Published:** 2019-09-05

**Authors:** Carina M. Hall, Sierra Jaramillo, Rebecca Jimenez, Nathan E. Stone, Heather Centner, Joseph D. Busch, Nicole Bratsch, Chandler C. Roe, Jay E. Gee, Alex R. Hoffmaster, Sarai Rivera-Garcia, Fred Soltero, Kyle Ryff, Janice Perez-Padilla, Paul Keim, Jason W. Sahl, David M. Wagner

**Affiliations:** 1 The Pathogen and Microbiome Institute, Northern Arizona University, Flagstaff, Arizona, United States of America; 2 U.S. Department of Agriculture, Animal and Plant Health Inspection Service, Veterinary Services, San Juan, Puerto Rico, United States of America; 3 Bacterial Special Pathogens Branch, Centers for Disease Control and Prevention, Atlanta, Georgia, United States of America; 4 Dengue Branch, Centers for Disease Control and Prevention, San Juan, Puerto Rico, United States of America; University of Texas Medical Branch, UNITED STATES

## Abstract

**Background:**

*Burkholderia pseudomallei* is a soil-dwelling bacterium and the causative agent of melioidosis. The global burden and distribution of melioidosis is poorly understood, including in the Caribbean. *B*. *pseudomallei* was previously isolated from humans and soil in eastern Puerto Rico but the abundance and distribution of *B*. *pseudomallei* in Puerto Rico as a whole has not been thoroughly investigated.

**Methodology/Principal findings:**

We collected 600 environmental samples (500 soil and 100 water) from 60 sites around Puerto Rico. We identified *B*. *pseudomallei* by isolating it via culturing and/or using PCR to detect its DNA within complex DNA extracts. Only three adjacent soil samples from one site were positive for *B*. *pseudomallei* with PCR; we obtained 55 isolates from two of these samples. The 55 *B*. *pseudomallei* isolates exhibited fine-scale variation in the core genome and contained four novel genomic islands. Phylogenetic analyses grouped Puerto Rico *B*. *pseudomallei* isolates into a monophyletic clade containing other Caribbean isolates, which was nested inside a larger clade containing all isolates from Central/South America. Other *Burkholderia* species were commonly observed in Puerto Rico; we cultured 129 isolates from multiple soil and water samples collected at numerous sites around Puerto Rico, including representatives of *B*. *anthina*, *B*. *cenocepacia*, *B*. *cepacia*, *B*. *contaminans*, *B*. *glumae*, *B*. *seminalis*, *B*. *stagnalis*, *B*. *ubonensis*, and several unidentified novel *Burkholderia* spp.

**Conclusions/Significance:**

*B*. *pseudomallei* was only detected in three soil samples collected at one site in north central Puerto Rico with only two of those samples yielding isolates. All previous human and environmental *B*. *pseudomallei* isolates were obtained from eastern Puerto Rico. These findings suggest *B*. *pseudomallei* is ecologically established and widely dispersed in the environment in Puerto Rico but rare. Phylogeographic patterns suggest the source of *B*. *pseudomallei* populations in Puerto Rico and elsewhere in the Caribbean may have been Central or South America.

## Author summary

The objective of this study was to examine the distribution and abundance of *Burkholderia pseudomallei* in the environment in Puerto Rico. *B*. *pseudomallei* is a microbe that lives in soil and causes the disease melioidosis. We conducted sampling around Puerto Rico to survey for the presence of *B*. *pseudomallei* in the environment. Of the 600 environmental samples collected, we isolated live *B*. *pseudomallei* from just two soil samples collected from the same site, which was in a region of the island where *B*. *pseudomallei* had never been previously reported. These results suggest *B*. *pseudomallei* is widely dispersed but rare in the environment in Puerto Rico. *B*. *pseudomallei* isolates from Puerto Rico are most closely related to other strains from the Caribbean. Caribbean strains are inside a larger group that contained all analyzed isolates from Central/South America, suggesting that *B*. *pseudomallei* populations in the Caribbean may have been introduced from Central or South America.

## Introduction

The *Burkholderia* genus is a group of diverse, primarily soil-dwelling, Gram-negative bacteria that have many strategies to survive and persist in soil, including acid tolerance [1] and intrinsic antibiotic resistance [2]. These species employ a wide variety of ecological strategies, including degradation of common pollutants, mutualistic relationships with plants, and also pathogenic relationships with plants, humans, and/or animals (3–10). The taxonomy of this genus remains incomplete and new species are regularly described [3–5]. The genus is commonly separated into two major phylogenetic groups: the *B*. *pseudomallei* complex (BPC), consisting of *B*. *pseudomallei* and its most closely related phylogenetic relatives, and the *B*. *cepacia* complex (BCC) [6]. The BCC includes a number of species that can be opportunistic pathogens of immunocompromised individuals, especially cystic fibrosis (CF) patients [7, 8]. Some other *Burkholderia* species are not assigned to either of these complexes, including the important plant pathogens *B*. *glumae* and *B*. *gladioli*; *B*. *glumae* causes bacterial panicle blight, a devastating disease in rice plants [9].

*B*. *pseudomallei* is the causative agent of the disease melioidosis and considered a Tier 1 Select Agent by the US Centers for Disease Control and Prevention (CDC) [2, 10]. Melioidosis can be contracted via cutaneous inoculation, inhalation, or ingestion, and can present with extremely varied symptoms [11]; these vague symptoms and diverse clinical presentations, along with culture-based diagnostic anomalies, make it difficult to properly diagnose in clinical settings [2, 12]. No vaccines against *B*. *pseudomallei* are currently available, making rapid detection and specific antibiotic treatment crucial for favorable outcomes in infected humans. Successful antibiotic treatment typically includes a strict and long regimen of intravenous antibiotics, such as ceftazidime or meropenem, for at least two weeks, followed by oral antibiotics, such as co-trimoxazole, for up to six months [2, 10]. However, treatment can be complicated by the fact that *B*. *pseudomallei* is intrinsically resistant to several clinically relevant antibiotics [13]. Importantly, other *Burkholderia* species that co-exist with *B*. *pseudomallei* in the environment are known to have an intrinsic resistance to other clinically relevant antibiotics, such as meropenem resistance in *B*. *ubonensis* [14], and thereby represent a possible source of similar resistance in *B*. *pseudomallei*.

*B*. *pseudomallei* has an “open genome” that can readily incorporate new genomic content via lateral gene transfer [15]. As a result, it has a relatively large accessory genome (i.e. the genomic features variable present among different *B*. *pseudomallei* strains) and a relatively small core genome (i.e. the genomic features present in all *B*. *pseudomallei* strains). The core genome is currently estimated at ~1,600 genes but will likely continue to decrease due to a process known as core genome decay, just as the accessory genome will continue to increase [6]. This is because, as additional *B*. *pseudomallei* genomes are generated from novel isolates, components previously identified as part of the core genome will be missing in some of the new genomes and completely novel components will also be identified, both of which increase the size of the accessory genome [6]. Genomic islands, often associated with tRNA sequences [16], contribute much of the genomic diversity observed in the *B*. *pseudomallei* accessory genome and some are hypothesized to contain virulence components [16, 17]. The adaptive potential of the large accessory genome in *B*. *pseudomallei* may be substantial, but remains poorly understood.

Determining where *B*. *pseudomallei* is present in the environment is crucial for understanding the potential risk to humans of acquiring melioidosis. This is because almost all infections with *B*. *pseudomallei* are independently acquired from the environment (27); human to human transmission of melioidosis is extremely rare [18]. *B*. *pseudomallei* has long been known to be endemic in tropical regions in northern Australia and Southeast Asia but the true global distribution appears to be much larger. Because melioidosis can be difficult to diagnose, it is possible that *B*. *pseudomallei* is also present in the environment in other regions of the world and causing human disease in these areas but going undetected [19].

**Fig 1:**
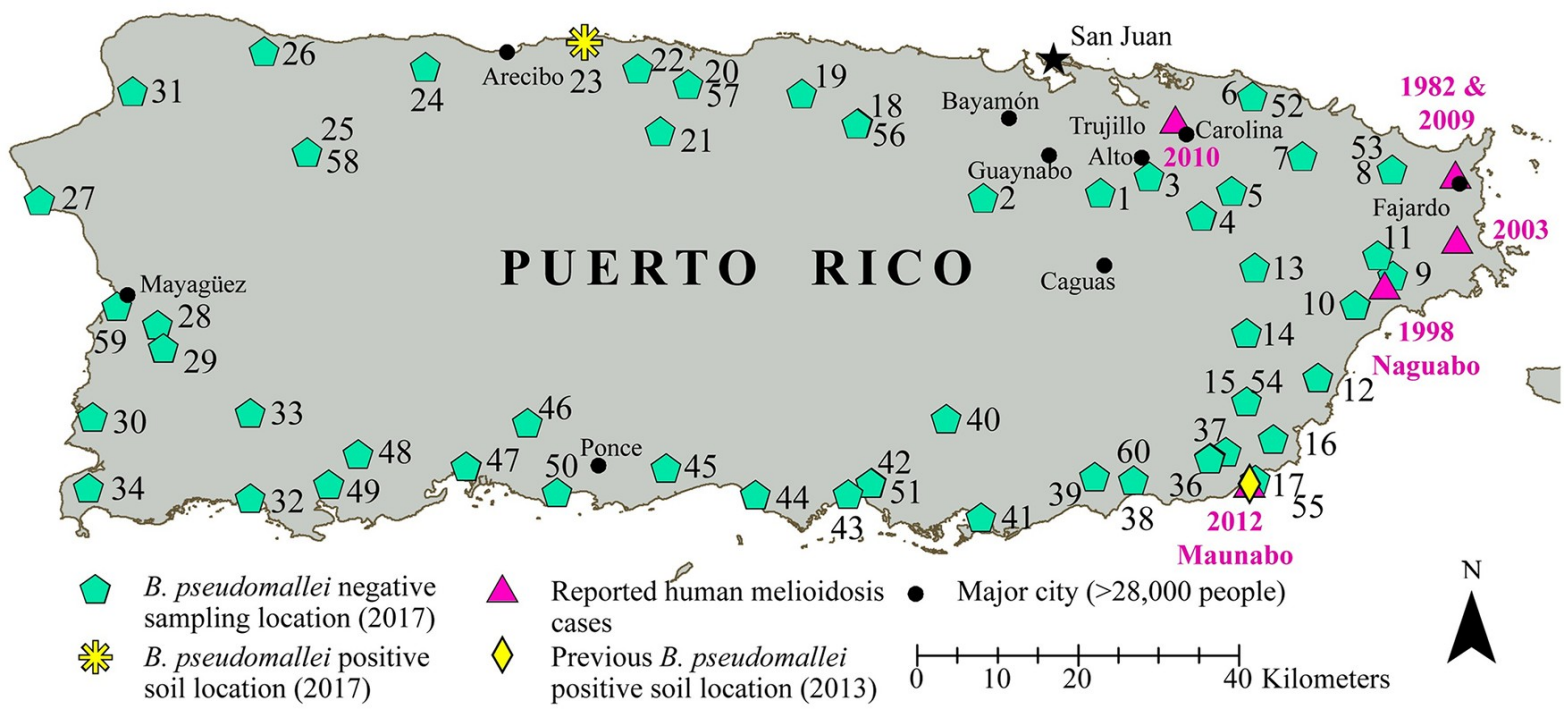
Locations in Puerto Rico where 1) environmental samples were collected in this study, 2) previous human melioidosis cases occurred, and 3) one previous *B*. *pseudomallei*-positive soil sample was collected. Site numbers are located next to the shapes indicating the 60 sampling locations from this study; soil samples were collected at sites 1–50 and water samples were collected at sites 51–60. The locations of past human melioidosis cases and the one previous *B*. *pseudomallei* positive soil location [20] are indicated. This map was created using ArcGIS software by Esri.

The majority of Puerto Rico experience a tropical rainforest climate (based on the Köppen climate classification), which is commonly associated with the presence of *B*. *pseudomallei* and human melioidosis cases have been previously reported from the island. Since 1982, there have been a total of seven reported human cases from Puerto Rico, all from the more populated eastern portion of the island (Fig 1) [20, 21]. A recent human melioidosis case from Puerto Rico, in 2012, occurred in the southeast municipality of Maunabo. Subsequent soil sampling in this region in 2013 resulted, for the first time, in the isolation of *B*. *pseudomallei* from the environment in Puerto Rico [20]. These previous human melioidosis cases (all but one with no travel history) and the isolation of *B*. *pseudomallei* from soil indicated that *B*. *pseudomallei* was present in the environment in Puerto Rico. The primary goal of this study was to gain a better understanding of the prevalence and geographic distribution of *B*. *pseudomallei* and other *Burkholderia* spp. in the environment in Puerto Rico. To achieve this goal, we conducted widespread soil and water sampling around the island and analyzed the samples using PCR and culture-based approaches to identify the presence of *B*. *pseudomallei* and other *Burkholderia* species.

## Methods

### Environmental sampling in Puerto Rico

Methods for environmental sampling were based upon international consensus guidelines for sampling for *B*. *pseudomallei* in the environment [22], with additional modifications developed by the Menzies School of Health Research in Darwin, Australia [23].

#### Site selection

In April 2017, we surveyed for *B*. *pseudomallei* in Puerto Rico by collecting and analyzing 600 environmental samples (500 soil and 100 water) from 50 soil sites and 10 water sites around the island. Puerto Rico received 130 mm of rain in March 2017 and 119 mm of rain in April 2017, which was 192% above the normal expected rainfall in March and 138% above the normal expected rainfall in April [24, 25]. At the start of this study, limited information was available regarding the presence of *B*. *pseudomallei* in the environment in Puerto Rico; a single positive soil sample was identified from a site associated with a previous melioidosis cases [20]. Because of this, we did not perform systematic sampling across the entire island but, rather, focused our sampling efforts on locations that we suspected would be most likely to harbor *B*. *pseudomallei*. Within many endemic regions, such as the tropical Top End of Australia [26] and Laos [27, 28], *B*. *pseudomallei* is found more often at lower elevations. For this reason, we focused our sampling efforts in lower elevation areas near the outer perimeter of Puerto Rico (Fig 1). In addition, in some endemic regions a greater abundance of *B*. *pseudomallei* has been documented from soils in agriculture lands as compared to non-agriculture lands [29, 30]. That said, other studies have documented that non-agriculture lands can also contain a high prevalence of *B*. *pseudomallei* [31, 32]. As a result, we targeted agricultural lands with farm animals, farming, and/or irrigation present. The 60 sites where we collected environmental samples included 53 sites located on agriculture lands, three sites located in natural reserves with little human impact, and four sites on public lands when nearby agriculture sites were not suitable for environmental sampling or were inaccessible. Permission was received from landowners to collect soil and/or water samples on their property and, when necessary, permits were obtained to collect soil and/or water samples from reserve lands. Because all previous reports of *B*. *pseudomallei* from humans and the environment in Puerto Rico originated in the eastern portion of the island, suggesting that it may be limited to that geographic region [33], we sampled more intensively in that region (Fig 1).

#### Soil sampling

At each soil collection site, separate samples were collected at a depth of 30cm below the surface from ten different holes spaced 2.5 meters apart along a single linear transect. Approximately 30g of soil from each sample was placed into a clean 50mL conical tube and stored in a covered insulated container in the shade to prevent direct UV exposure. Digging tools and other equipment were cleaned and decontaminated between samples to prevent cross contamination between samples and sites: equipment was first scrubbed with water to remove any large soil particles and then sprayed with 70% isopropyl alcohol to decontaminate. Soil pH for each sample was measured using a calibrated handheld pH meter: approximately 10g of soil was placed into a 50mL conical tube containing 40mL of DI water and the soil/water mixture was shaken by hand until the mixture was well homogenized and, following a 1-minute incubation at ambient temperature, the pH was measured using an Oakton EcoTestr pH 2 Waterproof pH Tester, with the pH reading recorded once the value stabilized.

#### Water sampling

At each water collection site, ten samples were collected along a single linear transect with samples collected approximately 2.5 meters apart, when possible. For each sample, 1L of water was collected into a Whirl-Pak bag, utilizing an extendable sampling pole when needed. At sites with flowing water, samples were collected near the water edge where there was less disturbance from the current. Water pH was measured from the first and last water samples at each site using a calibrated Oakton EcoTestr pH 2 Waterproof pH Tester and the pH readings were recorded once the pH values stabilized. Water samples were stored out of direct sunlight at ambient temperature until ready for filtering. The water samples were filtered in Puerto Rico using a Sartorius water filtration device that consisted of a Combisart 3 branch manifold with 250mL sterile funnels containing Microsart filters (cellulose nitrate, 47mm diameter, 0.2μM pore size) and a Microsart EJet pump. Sterile Minisart syringe filters (25mm, 0.2μm PTFE) were attached to each branch on the apparatus for sterile venting. Each water sample was split into three parts so that each water sample was filtered through three different filters. Once a water sample was completely filtered, all three filters were collected using sterile forceps and placed into a single sterile 50mL conical tube. All soil samples and filters from water samples were shipped at ambient temperatures to Northern Arizona University (NAU) and stored in the dark at ambient temperature until processed.

#### Culturing *Burkholderia* species

All culturing activities occurred at NAU and were conducted within containment using a biosafety cabinet in a BSL-2 laboratory, or in a Select Agent BSL-3 facility when *B*. *pseudomallei* was identified; all requisite entities were notified after detection of *B*. *pseudomallei*. Culturing followed international consensus guidelines [22] with specific modifications as previously described [34]. In short, 20g of soil was aseptically placed into a sterile 50mL bio-reaction tube with a hydrophobic membrane cap for venting (CellTreat, Pepperell, MA) that contained 20mL of sterile water. The soil and water mixture was vortexed until homogenized while using Parafilm to cover the tube to prevent the filter cap from becoming saturated. The samples were then incubated for 48 hours at 37°C while shaking at a speed to achieve aeration. After 48 hours, the shaking was stopped and the samples were allowed to settle for one hour before handling. A glycerol stock was created by adding 1mL of the top layer of the soil/water solution into a 2mL cryovial containing 500μL of concentrated Luria-Bertani (LB) broth and glycerol, resulting in a final concentration of 1 x LB broth with 20% glycerol. This glycerol stock was stored indefinitely at -80°C to serve as a backup culturing reserve. Then, 10μL from the top layer of the water/soil solution was plated on a small portion of half of an Ashdown’s agar plate (containing 4mg/mL of gentamycin) and streaked for isolation using the rest of the same half of the plate. 100μL of the top layer of the water/soil solution was plated on the other half of the Ashdown’s agar plate.

An enrichment culture in Ashdown’s broth was then initiated to favor growth of *B*. *pseudomallei* and other *Burkholderia* spp. 10mL of the soil/water solution was transferred into a new 50mL filter cap conical tube containing 30mL of Ashdown’s broth (containing 50mg/L of colistin). For water samples, all three filters from one water sample were added to a 50mL filter cap conical tube containing 30mL of Ashdown’s broth (containing 50mg/L of colistin). Both soil and water samples in Ashdown’s broth were incubated at 37°C for seven days while shaking at 130rpm. During incubation, we sampled repeatedly from these enriched broth cultures to test for the presence of *B*. *pseudomallei*. First, 3mL of the Ashdown’s broth incubated 2–5 days was placed into a new tube and pelleted at 3,750 x g for 10 minutes. The supernatant was removed and the pellet was stored at -20°C until ready for DNA extraction (described below). Second, we removed 10μL and 100μL from the top layer of the Ashdown’s broth to culture onto a new Ashdown’s agar plate after both two and seven days of incubation. Each of the above Ashdown’s plates was examined after 48 hours of incubation at 37°C for any colonies of interest. A sub-culture was performed onto a new Ashdown’s agar plate if a colony had an appearance similar to *B*. *pseudomallei*: lavender to purple colonies, dry, slightly textured, with a raised dome or fried-egg morphology, and dimpled/wrinkled centers [35–37]. All sub-culture plates were incubated at 37°C for 48 hours until DNA extraction.

#### Meropenem susceptibility

Meropenem susceptibility was determined for 11 *B*. *ubonensis* strains and two *B*. *pseudomallei* strains (Bp9039 and Bp9110) using Etests (bioMérieux, Durham, NC). The meropenem minimal inhibitory concentration (MIC) for six of the 11 *B*. *ubonensis* strains was previously described [14] and all meropenem Etests were conducted using the same methods described in that previous work. Briefly, an isolate was grown with the Etest on Mueller Hinton agar for 24 hours at 37°C and then zones of inhibition were recorded.

#### Detection of *B*. *pseudomallei* and *Burkholderia* spp

PCR was used for the detection of *B*. *pseudomallei* and *Burkholderia* spp. in DNA extracts taken from enrichment cultures of Ashdown’s broth. The microbial community in the Ashdown’s broth was screened for the presence of *B*. *pseudomallei* using a real-time PCR assay that targets *orf2* in the type three secretion system 1 (TTS1) cluster of *B*. *pseudomallei* [38]. DNA was extracted from the stored pelleted broth using QIAamp Fast DNA Stool Mini Kit (QIAGEN, Germantown, MD) following the manufacturer’s instructions, after first re-suspending the broth pellet with 1mL of the InhibitEX Buffer. All Ashdown’s broth DNA extractions were diluted to 1/30 using molecular grade water. As a quality control step, the DNA extractions were first screened with a real-time SYBR PCR assay using published conditions [39] of universal 16S rRNA primers [40]. If the 16S PCR confirmed that the DNA extractions were successful, then the DNA dilutions were screened in duplicate with a TaqMan assay targeting TTS1 to detect the presence of *B*. *pseudomallei* DNA [38]. Any broth extractions that were initially positive for *B*. *pseudomallei* with the TTS1 assay were then screened again in triplicate using the same assay; all real-time TaqMan assays were run on ThermoFisher 7900 instruments. Any sample in which the Ashdown’s broth community had a signal for *B*. *pseudomallei* resulted in even more intensive culturing efforts for that particular soil sample, all within NAU’s Select Agent BSL-3 facility. These additional culturing efforts included plating stored glycerol stocks that were created from the soil/water solution (before Ashdown’s broth inoculation) onto fresh Ashdown’s agar plates. All Ashdown’s agar plates were heavily sub-cultured for colonies with morphologies of interest (see above). In some cases, the culturing process was also repeated with a new aliquot of 20g of raw soil.

#### Single sample *B*. *pseudomallei* isolates

To investigate the diversity of *B*. *pseudomallei* within single soil samples (23–07 and 23–09), multiple suspected *B*. *pseudomallei* colonies were selected from the Ashdown’s plates created from the soil glycerol stock. All confirmed *B*. *pseudomallei* isolates were whole genome sequenced (see below).

#### Other *Burkholderia* spp

A crude DNA extraction followed by an assay that is largely specific to *Burkholderia* was performed to determine if a colony morphology of interest was a *Burkholderia* spp. DNA was extracted from sub-cultured colonies from the Ashdown’s agar plates using a 5% Chelex-100 heat soak method [41, 42]. Using standard PCR, these DNA extracts were screened with *Burkholderia* specific primers BUR3 [43] and BUR5 [35], which target a 365bp region of the *recA* gene; PCR conditions were as previously described [34]. The PCR product was run on an agarose gel and, if a band was present at the target size (365bp), Sanger sequencing was conducted using methods for both procedures as previously described [34]. Resulting amplicon sequences were searched using NCBI _BLAST_ (https://blast.ncbi.nlm.nih.gov/Blast.cgi) to identify isolates to genus. These approaches also identified other soil-dwelling genera from our samples, such as *Cupravidus*, *Delftia*, *Pseudomonas*, and *Ralstonia*; however, only *Burkholderia* spp. are reported herein.

All molecularly-confirmed *B*. *pseudomallei* and other *Burkholderia* spp. were processed for long-term storage and whole genome sequencing. A total of three isolation streaks were performed using Ashdown’s agar plates. A single colony was then selected to produce a lawn on a Luria-Bertani (LB) agar plate that was used to create glycerol stocks that are stored indefinitely at -80°C for future use. High quality genomic DNA for whole genome sequencing was extracted from purified isolates using a DNeasy Blood & Tissue Kit (QIAGEN, Germantown, MD), following the manufacturer’s instructions. Prior to sequencing, DNA extractions were screened again with the TTS1 or *recA* PCR assays described above to confirm species identification. Controls were used for all real-time and standard PCR reactions. These included DNA from a reference *B*. *pseudomallei* strain (K96243) as a positive control, and water for no-template controls (NTCs).

#### Whole genome sequencing

DNA library construction for whole-genome sequencing (WGS) was performed using KAPA Hyper Prep Kits (Roche, Pleasanton, CA) for Illumina NGS platforms per manufacturer’s protocol, with double-sided size-selection performed after sonication. Dual indexing was used [44] with adapters and 8bp index oligos from IDT (Integrated DNA Technologies, San Diego, CA) used in place of those supplied in the KAPA kit. The final libraries were quantified on an Applied Biosystems QuantStudio 7 Flex Real-Time PCR System (Invitrogen, ThermoFisher) using the KAPA SYBR FAST ROX Low qPCR Master Mix (Roche, Pleasanton, CA) for Illumina platforms. The libraries were then pooled together at equimolar concentrations and quality was assessed with a Bioanalyzer DNA 1000 chip (Agilent Technologies, Santa Clara, CA). Final quantitation by qPCR preceded sequencing of the final library. Final pools were sequenced on the Illumina MiSeq platform (Illumina, San Diego, CA) with the 600-cycle v3 kit for 250 cycles.

#### Genome assembly

Genomes were assembled with SPAdes v3.11.0 [45]. Contigs that showed an anomalously low depth of coverage or aligned to known contaminants based on BLASTN [46] alignments against the GenBank [47] nt database were manually removed.

#### MLST

Multi-locus sequence typing (MLST) was performed *in silico* on the *B*. *pseudomallei* and other *Burkholderia* spp. genomes, respectively, using information from the existing MLST typing scheme for *B*. *pseudomallei* [48] and the existing MLST typing scheme for the *B*. *cepacia* complex [49]. All MSLT data, including novel allele sequences and sequence types (STs), were submitted to PubMLST databases, either the *B*. *pseudomallei* MLST database (https://pubmlst.org/bpseudomallei/) or *B*. *cepacia* complex MLST database (https://pubmlst.org/bcc/) [50]. Novel STs found in this study are presented in S1 Table.

#### SNP calling and phylogenetics

To construct a *Burkholderia* spp. phylogeny (not including *B*. *pseudomallei*), genome assemblies from *Burkholderia* spp. collected in this study (S1 Table), along with a set of reference *Burkholderia* spp. genomes (S2 Table), were aligned against the reference *B*. *pseudomallei* genome K96243 [51] using NUCmer [52]; single nucleotide polymorphisms (SNPs) were then identified using NASP [53]. To construct a *B*. *pseudomallei* only phylogeny, raw reads were aligned against K96243 with BWA-MEM [54] and SNPs were called with the UnifiedGenotyper method in GATK [55]. For both methods, SNPs that fell within duplicated regions, based on NUCmer reference self-alignment, were filtered from downstream analyses. Maximum likelihood phylogenies were inferred from concatenated SNP alignments using IQ-TREE v1.6.1 [56] and 1,000 bootstrap replicates. Additionally, to estimate Bayesian time to most recent common ancestor (TMRCA) for each separate *B*. *pseudomallei* chromosome, individual SNP matrices and phylogenies were generated that included genome assemblies from eight *B*. *pseudomallei* isolates from Puerto Rico (*n* = 7) and Trinidad (*n* = 1) with three *B*. *pseudomallei* isolates from Martinique (S2 Table) to serve as an outgroup. Although both chromosomes 1 and 2 were analyzed separately, no molecular clock signal was detected for chromosome 2. As such, only chromosome 1 was used for all subsequent molecular clock analyses.

#### Comparative genomics

A pan-genome analysis was performed on all new *B*. *pseudomallei* genomes generated in this study (S3 Table) using the Large scale BLAST score ratio (LS-BSR) pipeline with a 0.95 BSR threshold [57] and the blat [58] alignment option. Coding regions that were variably conserved were extracted from the matrix and visualized with the Interactive Tree of Life [59]. Similar approaches were used to determine, within the set of reference genomes (S2 Table), the presence/absence in the reference genomes of coding regions that were variably conserved in the new *B*. *pseudomallei* genomes from Puerto Rico (S3 Table).

#### Genomic island identification

From the BSR results, we identified regions that were variably conserved in the genomes of *B*. *pseudomallei* isolates obtained from site 23 but were absent from other, geographically diverse *B*. *pseudomallei* genomes. From the NCBI PGAP annotation, we identified coding and flanking regions that were associated with identified genomic islands. To screen for the presence of these genomic islands in other *Burkholderia* spp., we screened all coding regions associated with genomic islands against all *Burkholderia* spp. genomes with LS-BSR, using a BSR threshold of >0.8 for presence; a lower threshold was used as diverse species were being screened. The structures of the genomic islands were visualized with the genoPlotR R package [60] using the PGAP annotation.

#### Root-to-tip regression analysis

Using the SNP alignment of chromosome 1 from the Puerto Rico sample subset, the program Gubbins [61] was used to test for and remove recombination, as this can confound divergence-dating analyses; all subsequent timing analyses were performed with the resulting data. A temporal signal was assessed in the program TempEst version 1.5.1 [62] using regression analysis implementing root-to-tip genetic distance as a function of the sample year. A measure of clocklike behavior was assessed using the determination coefficient *R*^*2*^, with the best-fitting root selected to maximize *R*^*2*^. To evaluate the significance of the regression analysis, we performed 10,000 random permutations of the sampling dates over the sequences [63].

#### Estimations of divergence times

A Bayesian relaxed molecular clock using tip dating was applied using the BEAST version 1.8.4 software package [64] to estimate the TMRCA for chromosome 1 of eight Puerto Rico isolates using three *B*. *pseudomallei* isolates from Martinique as an outgroup. The best nucleotide substitution model was inferred using the Bayesian information criterion and MEGA7 software [65]. BEAST analysis was run with a correction for invariant sites by specifying a Constant Patterns model in the BEAST xml file. A “path and stepping stone” sampling marginal-likelihood estimator was used to determine the best-fitting clock and demographic model combinations [66]. The log marginal likelihood was used to assess the statistical fits of 10 clock and demographic model combinations. Four independent chains of 750 million iterations each were run for the best clock and demographic model combination. Convergence among the four chains was confirmed in the program Tracer (version 1.6.0) [67]. Molecular clock and demographic combination testing was performed using strict and relaxed molecular clocks in combination with five demographic priors. Model combinations that failed to converge were discarded.

#### YLF and ITS typing

The presence/absence of the *Yersinia*-like fimbrial (YLF) gene [68] in all new *B*. *pseudomallei* strains collected in this study was determined by LS-BSR (accession sequence YP_110141.1). In addition, all new isolates were typed for length polymorphisms in the 16S-23S internal transcribed spacer (ITS) [69] by LS-BSR [accession sequences type C (FJ981718.1), type G (FJ981723.1), and type E (FJ981706.1)].

#### Data accession

All short reads and genome assemblies were submitted to GenBank under BioProject accession PRJNA451205. Accession numbers for individual genomes are shown in S1 and S2 Tables.

## Results

### *B*. *pseudomallei* was found in the environment at one new location in Puerto Rico

Our broad environmental survey in April 2017 resulted in the identification of *B*. *pseudomallei* from soil samples collected at only one new location, in the northern municipality of Arecibo (Fig 1). Just three of the DNA extracts obtained from the 600 enriched Ashdown’s broth samples contained *B*. *pseudomallei* DNA; *B*. *pseudomallei* was not detected in any of the 100 water samples. The positive samples originated from three adjacent soil samples collected from a single sampling site (site 23; Fig 1). Site 23 was located on a farm in the municipality of Arecibo where swine, goats, chickens, and cattle were present. DNA extractions from three Ashdown’s broth samples (23–07, 23–08, and 23–09) tested positive with the *B*. *pseudomallei*-specific TTS1 PCR assay (run in triplicate). It is important to note that the presence of *B*. *pseudomallei* DNA in these complex DNA samples did not definitively indicate that live *B*. *pseudomallei* was present in the enriched broth samples or the original soil samples. However, this information allowed us to refocus our culturing efforts on these samples to attempt to isolate *B*. *pseudomallei*. Two of the three soil samples did yield *B*. *pseudomallei* cultures: *B*. *pseudomallei* isolate Bp9039 was obtained from soil sample 23–07 on the first culturing attempt, and a second round of culturing from another 20g of soil from sample 23–09 yielded *B*. *pseudomallei* isolate Bp9110. Additional rounds of culturing yielded other isolates from 23–07 and 23–09 (S3 Table) but no *B*. *pseudomallei* isolates were ever obtained from soil sample 23–08 despite a positive *B*. *pseudomallei* DNA signal from the Ashdown’s broth extraction and multiple attempts at culturing; this is not an uncommon occurrence when surveying for *B*. *pseudomallei* in the environment [22]. *B*. *pseudomallei* isolates Bp9039 and Bp9110 were both susceptible to meropenem; other collected *B*. *pseudomallei* isolates were not tested.

### pH of environmental samples

The pH of the soil samples collected around the island varied greatly from highly acidic to highly alkaline, whereas the water samples varied from a neutral pH to a highly alkaline pH (S4 Table). All soil samples across all sites had an average pH of 7.3 with a range of 3.2–11 and all water samples from all sites had an average pH of 7.8 with a range of 6.8–10.2. The three *B*. *pseudomallei*-positive soil samples from site 23 (07, 08, and 09) yielded pH values of 4.9, 4.9, and 5.1, respectively. The pH of soil samples 01–06 from site 23 were 7.6, 7.2, 7.0, 5.1, 6.2, and 6.8, respectively; the pH of soil sample 10 from site 23 was 4.9. No association between soil pH and the occurrence of *B*. *pseudomallei* was detected in this study, which was not unexpected given the very small number of *B*. *pseudomallei*-positive soil samples (*n* = 3).

### *B*. *pseudomallei* isolates from this study are similar to previous isolates from Puerto Rico

**Fig 2:**
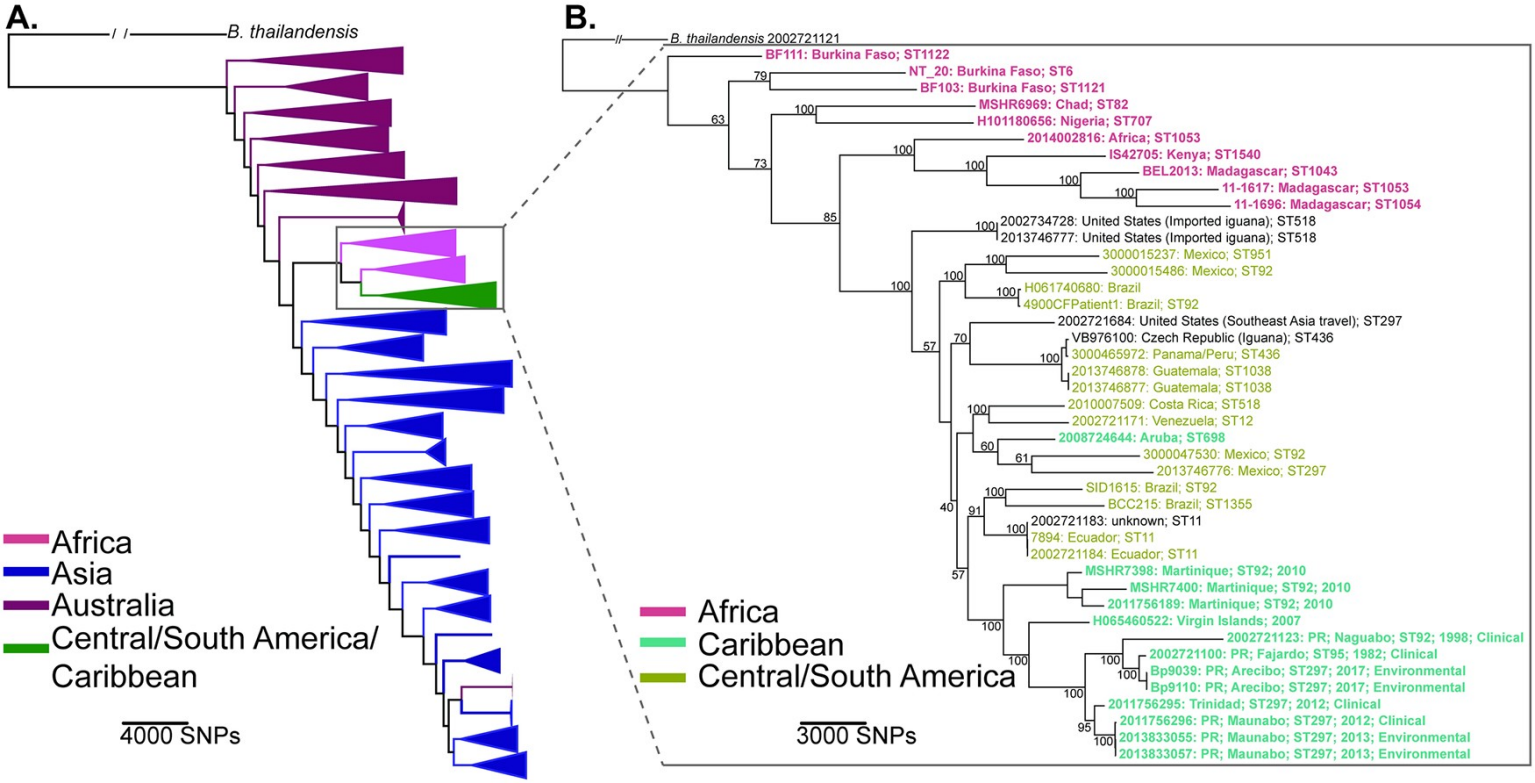
*Burkholderia pseudomallei* global whole genome phylogeny. Maximum-likelihood phylogeny of 414 globally-diverse *B*. *pseudomallei* isolates rooted with a *B*. *thailandensis* isolate; bootstrap values are reported on nodes. Two of the 55 *B*. *pseudomallei* isolates obtained from site 23 in this study are included (Bp9039 and Bp9110). (A) All 414 *B*. *pseudomallei* genomes with nodes collapsed. (B) Expanded nodes within the monophyletic group containing all included isolates (*n* = 44) from Africa, Central and South America, Mexico, and the Caribbean.

Within a core genome phylogeny of 414 globally diverse *B*. *pseudomallei* isolates (Fig 2, S2 Table), two *B*. *pseudomallei* isolates from the municipality of Arecibo (Bp9039 and Bp9110) are highly similar to each other (both isolates were assigned to MLST ST297) and are nested within a large monophyletic group that contains all included *B*. *pseudomallei* isolates from other locations in the Caribbean, Central and South America, Mexico, and Africa (Fig 2, panel B). Within this larger group, the new isolates from Puerto Rico and the previous *B*. *pseudomallei* isolates obtained from Puerto Rico form a distinct subgroup together with one isolate from Trinidad. Within that subgroup are two distinct lineages: one including the new environmental isolates from Arecibo with some previous clinical isolates from Puerto Rico, and a second lineage including the previous environmental isolates collected near the 2012 clinical isolate from Maunabo, as well as the one 2012 clinical isolate from Trinidad.

The root-to-tip regression analysis identified weak clocklike behavior among the Puerto Rico and Martinique sample set with an *R*^*2*^ value of 0.1201. However, the positive regression slope indicates molecular clock analysis is still reliable for mutation rate estimation [70]. The best-fitting nucleotide substitution model implemented based on MEGA7 model testing was GTR. The 10,000-date randomization permutation testing produced a p-value of 0.184, suggesting that the *R*^*2*^ value produced in the root-to-tip regression analysis was not statistically different than random chance. Stepping-stone and path-sampling analyses did not show marked differences; a relaxed clock and extended Bayesian skyline plot was selected as the model combination for this analysis. The BEAST timing analysis had a mean estimate of the year 1950 (95% HPD, 1923 to 1975; S1 Fig) for the TMRCA of chromosome 1 for the eight *B*. *pseudomallei* isolates from Puerto Rico and Trinidad. The evolutionary rate was estimated at 5.01E-6 (95% HPD, 2.81E-6 to 8.28E-6) for all eight samples and the Martinique outgroup. This is in contrast to another study that found an evolutionary rate of 1.80E-6 (95% HPD, 1.36E-6 to 2.66E-6) for chromosome 1 for multiple *B*. *pseudomallei* isolates from the Americas [71].

### *B*. *pseudomallei* genomic diversity observed from a single sampling site

We observed fine-scale genomic diversity among multiple *B*. *pseudomallei* isolates obtained from a single sampling site and even from a single soil sample. A total of 55 *B*. *pseudomallei* isolates were isolated from two soil samples at site 23, with 50 isolates from soil sample 23–07 and five isolates from soil sample 23–09 (S3 Table). It is important to note that these isolates were obtained from enriched culture medium so it is possible that less than 55 individual *B*. *pseudomallei* cells were present in the original samples. All 55 isolates were similar in regards to being assigned to the same ST (297) and to ITS type G; they all also contained the YLF gene cassette. However, variation was still observed among these strains in the core genome phylogeny (48 unique SNP genotypes were identified among the 55 isolates). There are three distinct clades (A-C) observed in the core genome phylogeny for these isolates, with isolates from soil sample 07 (*n* = 50) assigning to all three clades, while all isolates from soil sample 09 (*n* = 5) assigned to just clade A along with two of the isolates from soil sample 07 (S2 Fig). Interestingly, there were two *B*. *pseudomallei* isolates from different soil samples (Bp9046-sample07 and Bp9110-sample09) that were very similar: these two strains exhibit no SNP differences in the core genome (S2 Fig).

**Fig 3:**
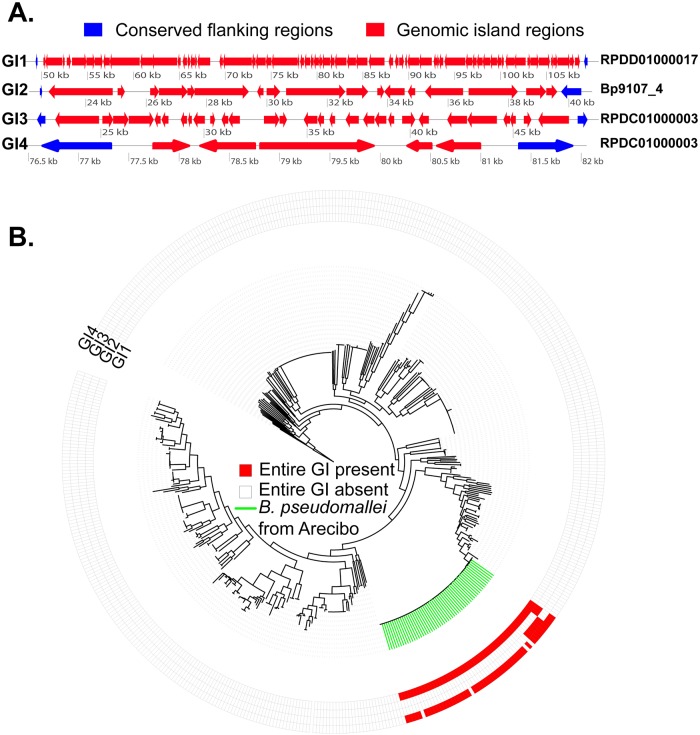
Genomic islands present in *B*. *pseudomallei* isolates from a single location in Puerto Rico. (A) Reveals the structure of the four novel *B*. *pseudomallei* genomic islands (GI1-4) that were discovered in this study; contig names are listed on the far right. The red arrows reflect *B*. *pseudomallei* genes found within the genomic islands and the blue arrows reflect conserved flanking regions commonly found in other *B*. *pseudomallei* strains. (B) Circular phylogeny with genomic islands mapped on the outside of the phylogeny. This phylogeny was constructed using the same 414 *B*. *pseudomallei* isolates used to generate Fig 2 plus the 53 additional *B*. *pseudomallei* isolates from site 23 that were not included in Fig 2.

We identified four distinct genomic islands (GI1-GI4) among the 55 *B*. *pseudomallei* isolates obtained from site 23 (Fig 3), and these contain a subset of genes not found in any other *B*. *pseudomallei* genomes. The insertion of these genomic islands appears to be associated with tRNA gene loci (S5 Table), which is similar to previous patterns described from *B*. *pseudomallei* [16]. GI1 (comprised of 61 genes; S5 Table) is conserved across all 55 of the *B*. *pseudomallei* isolates from site 23, whereas GI2 (comprised of 15 genes; S5 Table), GI3 (comprised of 29 genes; S5 Table), and GI4 (comprised of 5 genes; S5 Table) are variably present among the 55 isolates from site 23 (Fig 3B). The accessory genome of the 55 *B*. *pseudomallei* isolates from site 23 is comprised of 58 genes: 49 (1–49; S2 Fig, S5 Table) are contained in GI2, GI3, and GI4 and nine others occur at different genomic locations (50–58; S2 Fig, S5 Table). GI2 is conserved among clade A isolates but not found in the other two clades, GI3 is conserved among clade B isolates but not found in the other two clades, and GI4 is conserved among clade B isolates and variably present in clade A and clade C isolates (S2 Fig). None of the four genomic islands were found in a complete form in 412 other globally diverse *B*. *pseudomallei* genomes that were examined, including the genomes of *B*. *pseudomallei* isolates obtained from other locations in Puerto Rico (Fig 3B, S5 Table), nor in the genomes of 781 other *Burkholderia* spp. isolates (S5 Table). A majority (*n* = 44) of the 61 genes within GI1 were found in at least one of the genomes of the 1,193 other *B*. *pseudomallei* and/or *Burkholderia* spp. isolates that were examined, but the other 17 genes in GI1 were only found in the site 23 isolates (S5 Table); none of the genes in GI2 were found in these other genomes (S5 Table). A majority of the genes within GI3 (25/29) and GI4 (4/5) were not found in any of the other 1,193 genomes, but all of the nine accessory genes that occurred outside of the genomic islands were found in other *B*. *pseudomallei* genomes and some were also found in the genomes of other *Burkholderia* spp. (S5 Table). Interestingly, it was more common for genes from GI1 and GI3 to be found in the genomes of other *Burkholderia* spp. than in the genomes of other, global *B*. *pseudomallei* isolates (S5 Table).

### Widespread environmental dispersal of many other *Burkholderia* species

A number of other *Burkholderia* species are widespread and common in both soil and water throughout Puerto Rico (S4 Table). A total of 686 sub-cultures were selected from Ashdown’s agar plates from 301 soil samples (collected from all 50 soil collection sites) and 77 water samples (collected from all 10 water collection sites). Of these sub-cultures, 129 were identified as members of the *Burkholderia* genus according to the sequence of a *recA* gene fragment. Most of the 129 *Burkholderia* isolates (*n* = 104) were isolated from 61 different soil samples (originating from 20 of the 50 soil sampling sites), with only 25 isolated from 22 different water samples (but originating from seven of the 10 water sampling sites). *Burkholderia* spp. were cultured from the environment in 21 of the 41 sampled municipalities within Puerto Rico. It is important to note that this does not indicate that there were not *Burkholderia* spp. present in the environmental samples collected at the other 20 municipalities, only that we did not successfully culture any *Burkholderia* spp. from environmental samples collected from those locations using our methods.

**Fig 4:**
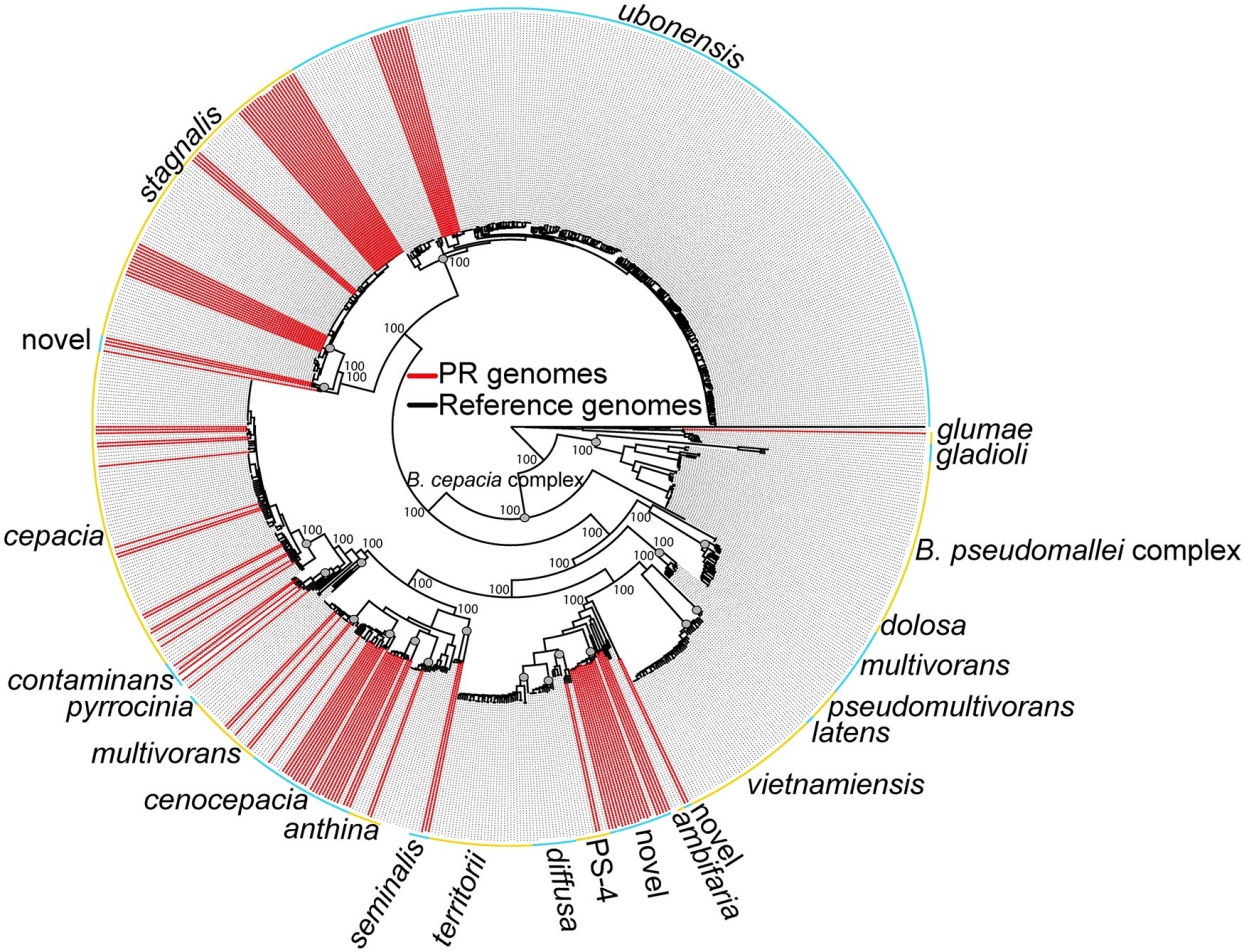
*Burkholderia* spp. whole genome phylogeny. Core genome maximum-likelihood phylogeny of 781 *Burkholderia* spp. isolates rooted with *Cupriavidus*. The 781 isolates include 129 isolates obtained from Puerto Rico in this study (indicated with red lines; S1 Table) and 651 publicly available *Burkholderia* spp. isolates (S5 Table). Bootstrap values are reported on nodes.

The 129 *Burkholderia* spp. isolates were identified in a whole genome phylogeny (Fig 4) as follows: *B*. *anthina* (*n* = 2), *B*. *cenocepacia* (*n* = 29), *B*. *cepacia* (*n* = 15), *B*. *contaminans* (*n* = 5), *B*. *glumae* (*n* = 1), *B*. *seminalis* (*n* = 2), *B*. *stagnalis* (*n* = 36), *B*. *ubonensis* (*n* = 11), and other unidentified novel *Burkholderia* spp. (*n* = 28) (S1 Table). A total of 332 novel MLST alleles were identified from the 129 isolates, resulting in 102 novel STs (S1 Table). All *Burkholderia* isolates cultured from this study belong to the *B*. *cepacia* complex with the exception of *B*. *glumae*, which is genetically distinct from both the BPC and BCC. The single *B*. *glumae* isolate was identified from a water sample collected in Patillas, Puerto Rico. *B*. *ubonensis* appears widespread throughout Puerto Rico, with 11 isolates obtained from five municipalities spread across the island [Barceloneta (*n* = 1), Cabo Rojo (*n* = 4), Ceiba (n = 4), Juncos (*n* = 1), and Maunabo (*n* = 1); S4 Table]. All 11 *B*. *ubonensis* isolates were resistant to meropenem (>32μg/mL) (S1 Table).

## Discussion

*B*. *pseudomallei* is ecologically established and widely dispersed in the environment in Puerto Rico but rare. It has now been isolated from soil samples from two regions of the island separated by >100 kilometers (Fig 1). Despite this widespread geographic distribution, it is also quite rare in the environment in Puerto Rico. Even with extensive sampling at 60 different soil and water sites located around the island (Fig 1), *B*. *pseudomallei* was detected at just one of the 50 soil sampling sites and was not detected at any of the 10 water sampling sites. The new location where 55 *B*. *pseudomallei* soil isolates were obtained is in the central northern municipality of Arecibo; to date, there have been no reports of human melioidosis nor collection of environmental *B*. *pseudomallei* isolates from this region. The only previously reported human melioidosis cases from Puerto Rico were reported from municipalities located on the eastern region of the island, and the same is true for the two environmental soil isolates (obtained from the same soil sample) previously reported from this region [20] (Fig 1). Of note, in this study we did not detect *B*. *pseudomallei* from environmental samples collected in the eastern portion of the island even though we sampled more extensively in this region because the previous human and environmental isolates were obtained there. This result is also suggestive of the overall rarity of *B*. *pseudomallei* in the environment in Puerto Rico.

*B*. *pseudomallei* also appears to be rare locally in the environment of Puerto Rico at sites where it is present. When *B*. *pseudomallei* was previously detected in southeastern Puerto Rico it was only isolated from one of 20 soil samples collected from a single neighborhood [20]. Similarly, at the one site where *B*. *pseudomallei* was isolated from soil in this current study it was only detected in three of the 10 soil samples collected at that site, and these three *B*. *pseudomallei*-positive soil samples were adjacent to one another (thereby separated by <5 m). These findings suggest a locally clumped distribution for *B*. *pseudomallei* in the environment in Puerto Rico, a pattern that has also been reported from highly-endemic regions, such as northeast Thailand [72]. In addition, it is possible that *B*. *pseudomallei* is also rare at the level of individual soil samples in Puerto Rico because we made multiple attempts to successfully isolate it from one of the soil samples (23–09) and we were never able to isolate it from another soil sample (23–08) despite multiple attempts and a PCR result that indicated that *B*. *pseudomallei* DNA was present in DNA extracted from that same soil sample. This is in contrast to patterns from highly endemic regions, such as Thailand and northern Australia, where hundreds of *B*. *pseudomallei* isolates can often be obtained from a single soil sample [31, 73, 74].

The overall rarity of *B*. *pseudomallei* in the environment in Puerto Rico may be due to unsuitable environmental conditions. A recent study [19] estimated global environmental suitability for *B*. *pseudomallei* based upon predicted models developed using location data from >22,000 documented human and animal cases, which were primarily from highly endemic settings in southeast Asia and northern Australia. In the western hemisphere, this analysis predicted high environmental suitability for *B*. *pseudomallei* in large areas of northern South America, portions of Central America and Mexico, and several small areas in the southern United States. In contrast, it predicted low environmental suitability for *B*. *pseudomallei* for most locations in the Caribbean with a few exceptions. One of those exceptions was along the northwest coast of Puerto Rico, including the location in the municipality of Arecibo where we isolated *B*. *pseudomallei* in this study. That said, it is important to note that this same model predicts low environmental suitability for *B*. *pseudomallei* for the rest of Puerto Rico, including the eastern portion of the island where the previous *B*. *pseudomallei*-positive soil sample was collected and all previous known human melioidosis cases occurred (Fig 1).

It seems likely that *B*. *pseudomallei* was introduced to Puerto Rico relatively recently, possibly from other locations in the Caribbean. A recent introduction of *B*. *pseudomallei* would provide another explanation for why *B*. *pseudomallei* is rare in the environment in Puerto Rico. Several previous phylogenomic studies of *B*. *pseudomallei* have all noted a consistent pattern in which isolates from the Americas cluster together on a single branch that emerges from the larger African clade. Thus, the leading hypothesis for the introduction of *B*. *pseudomallei* to the Americas from Africa suggest that it occurred via the transatlantic human slave trade in the 16^th^-19^th^ centuries [17, 71, 75, 76]; molecular clock estimates in one of these studies support this proposed timeline [71]. Our phylogenetic results are consistent with this pattern of strains from the Americas forming a monophyletic clade that is nested within a larger clade containing all known *B*. *pseudomallei* isolates from Africa (Fig 2) but also provide further insights because we included additional strains from the Caribbean and other locations from the Americas. Within the monophyletic clade from the Americas, we found that all isolates from the Caribbean, with the exception of one isolate from Aruba, group together in a smaller monophyletic clade, and all of the seven known isolates from Puerto Rico grouped together in a smaller clade with one isolate from Trinidad. The molecular clock estimates from this study support a recent introduction of *B*. *pseudomallei* to Puerto Rico, within the last 70 years (S1 Fig, S2 Table). Together, these findings suggest that *B*. *pseudomallei* may have been first introduced to other regions of the Americas from Africa and then, more recently, was introduced to the Caribbean from these other regions of the Americas. Because isolates from Puerto Rico group together within a larger clade containing all but one of the other isolates from Caribbean, it is tempting to suggest that *B*. *pseudomallei* was introduced to Puerto Rico from other locations in the Caribbean. However, it is important to note that all of these ideas are based upon analysis of currently available *B*. *pseudomallei* isolates. As there are numerous countries where *B*. *pseudomallei* is thought to occur but has not yet been detected, especially in the western hemisphere [19], the global phylogeographic patterns of *B*. *pseudomallei* will almost certainly change as additional isolates are obtained from new locations and sequenced. In particular, more environmental sampling is necessary to better understand the occurrence and spread of *B*. *pseudomallei* in the Caribbean.

All included isolates from Puerto Rico, together with a single isolate from Trinidad, share a recent common ancestor in the global phylogeny (Fig 2), which is suggestive of a single introduction to Puerto Rico. In addition, the new *B*. *pseudomallei* soil isolates from Arecibo, the two previous soil isolates from Maunabo, and the previous human isolates from Maunabo have all been assigned to ST297, as has the human isolate from Trinidad. This pattern is not unexpected as ST297 in *B*. *pseudomallei* is typically associated with isolates from the Western Hemisphere [75]. This overall lack of diversity is in contrast to patterns observed in highly-endemic settings, such as northeast Thailand and northern Australia, where high levels of genetic diversity are observed at multiple spatial scales, including among multiple isolates obtained from single soil samples, using multiple genotyping approaches, including MLST, mutli-locus variable number tandem repeat analysis, and pulse field gel electrophoresis [31, 77]. That said, there are also two distinct lineages among the Puerto Rican isolates and one of these lineages contains two human isolates from Puerto Rico that were assigned to ST92 and ST95 [20] (Fig 2). Thus, it is also plausible that *B*. *pseudomallei* has been introduced to Puerto Rico multiple times. Again, additional environmental sampling in the Caribbean, including Puerto Rico, is needed to better understand these patterns.

As an alternative to the hypothesis of human-mediated dispersal, *B*. *pseudomallei* may have been introduced to Puerto Rico via hurricanes or other extreme weather events. In Australia and Asia, *B*. *pseudomallei* can become aerosolized during extreme weather events like cyclones (i.e., hurricanes), leading to subsequent increases in human disease events [78–80]. The predominate path of Atlantic hurricanes is generally from the east-southeast to the west-northwest, essentially directly through the Caribbean [81], which would be consistent with hurricanes dispersing *B*. *pseudomallei* to Puerto Rico from other locations to the southeast of it in South America or other regions of the Caribbean. Long distance dispersal of *B*. *pseudomallei* during extreme weather events also offers potential explanations for several other patterns observed in the phylogeny (Fig 2). For example, the single isolate from Trinidad, from a 2012 clinical case, clusters together with a 2012 clinical isolate from Puerto Rico (and two soil isolates collected in 2013 near the residence of the 2012 Puerto Rico case) rather than with isolates from more nearby locations in South America. In addition, a 2012 clinical isolate from Aruba clusters together most closely with isolates from Mexico and Central and South America, rather than with other isolates from the Caribbean (Fig 2). Of note, the 2012 Atlantic hurricane season was more active than normal, including 10 different hurricanes [82].

The four unique genomic islands identified from the genomes of *B*. *pseudomallei* isolates from site 23 may be indicative of adaptation to local ecological conditions. Identifying novel genomic components from new *B*. *pseudomallei* genomes is not at all unexpected as the accessory genome of this species is quite large and continues to grow as more isolates are sequenced [6], likely because this species has an “open genome” that can readily acquire new genomic content via lateral gene transfer [15]. However, what is striking is that the four genomic islands, as well as a majority of the genes within them, are only found in *B*. *pseudomallei* isolates from site 23 and not in other *B*. *pseudomallei* isolates or isolates from other *Burkholderia* spp. (S2 Table). Given the almost complete absence of these genomic islands in globally diverse isolates of *B*. *pseudomallei*, the genes contained within these genomic islands may have been obtained locally from other soil dwelling species as a means of adapting to fine-scale environmental conditions. In some cases, other *Burkholderia* spp. also shared a portion of these genomic islands, providing a potential source of these accessory genes. However, over half of the accessory genes found in the *B*. *pseudomallei* isolates from site 23 were completely absent from all of the other global *B*. *pseudomallei* and *Burkholderia* spp. genomes that were examined (S2 Table), suggesting that the source of many of these accessory genes may be species outside the *Burkholderia* genus that co-occur in the soil. Additional studies of the accessory genome of multiple *B*. *pseudomallei* isolates obtained from soil samples collected across small spatial scales will be important for yielding new insights into the possibility of the acquisition of new accessory genes representing a mechanism for *B*. *pseudomallei* to adapt to local ecological conditions.

Other diverse *Burkholderia* spp. are widespread in the environment in Puerto Rico and may be the source of some of the unique accessory genes in the *B*. *pseudomallei* isolates from site 23 in the municipality of Arecibo. Indeed, a number of the novel *B*. *pseudomallei* genes present in the four genomic islands described here were also identified from other *Burkholderia* spp. (S5 Table). Although there was no evidence of other members of the BPC, such as *B*. *thailandensis*, *B*. *oklahomensis*, or *B*. *humptydooensis*, being present in Puerto Rico, many different *Burkholderia* spp. from the BCC were isolated from both soil and water in Puerto Rico, including some potentially novel species (S4 Table; Fig 4). Due to the large number of novel BCC MLST alleles and STs identified among these BCC isolates, it appears that Puerto Rico harbors many unique and diverse BCC strains that have yet to be classified. And it is important to note that our survey almost certainly provides a limited understanding of the true diversity of *Burkholderia* spp. present in the environment in Puerto Rico as we only examined *Burkholderia* species that were capable of growing on Ashdown’s selective medium. Horizontal gene transfer from these other diverse and widespread *Burkholderia* spp. may facilitate adaptation of *B*. *pseudomallei* to local environmental conditions in Puerto Rico and elsewhere. One such concern is the transfer of intrinsic antibiotic resistance to clinically relevant antibiotics, such as the potential transfer of the meropenem resistance observed in *B*. *ubonensis*, to *B*. *pseudomallei*.

Overall, *B*. *ubonensis* was quite widespread in Puerto Rico: we isolated it from soil samples collected from five locations in the southwest, southeast, eastern, and north central portions of the island; we did not isolate it from any water samples (S4 Table). *B*. *ubonensis* has been previously described from the environment only from countries where *B*. *pseudomallei* is highly endemic, including Australia, Malaysia, Thailand, and Papua New Guinea [6]. To our knowledge, the *B*. *ubonensis* strains collected from Puerto Rico in this study are the first instance of this species being isolated from the environment in the Caribbean and the western hemisphere [14]. However, our results from Puerto Rico suggest that it may be widespread in the Caribbean and elsewhere in the western hemisphere. Previous studies have found that *B*. *ubonensis* is the most common BCC species to be co-isolated with *B*. *pseudomallei* [36]. The first isolates from Puerto Rico had a distant phylogenetic relationship to isolates from Australia [14]. Interestingly, we did not find any evidence of *B*. *ubonensis* from the municipality of Arecibo where *B*. *pseudomallei* was isolated. However, we did find *B*. *ubonensis* from the municipality of Maunabo, where *B*. *pseudomallei* was previously found in the soil in 2013. We found that all of the *B*. *ubonensis* strains that we collected from Puerto Rico (11 of 11) were resistant to meropenem (S1 Table), an antibiotic used to treat patients with advanced melioidosis, such as sepsis [2]. A previous study investigating the meropenem resistance of *B*. *ubonensis* found that 21% of tested strains from Australia and 67% of tested strains from Thailand were meropenem resistant [14].

Until this study, *B*. *glumae*, a USDA-APHIS regulated plant pathogen, had not been described from Puerto Rico since 2004, when it was identified from an onion plant [83]. We detected *B*. *glumae* in a water sample from the municipality of Patillas (S4 Table). This plant pathogen causes bacterial panicle blight and can be devastating to various types of crops, including rice [9]. As a result, the appropriate regulating agencies within Puerto Rico and the United States were notified of the presence of *B*. *glumae* at this location. The knowledge of the presence of this plant pathogen at this location can serve as vital information when investigating potential crop infestations.

### Conclusions

Widespread environmental surveys for *B*. *pseudomallei* in the environment in Puerto Rico identified the pathogen from soil samples collected in a region of Puerto Rico from which it had never been previously detected in the environment or in humans. This study demonstrates that although *B*. *pseudomallei* is present in the environment in several widespread locations in Puerto Rico, it is also rare. Given how rare it is in the environment, *B*. *pseudomallei* does not appear to pose a large public health risk in Puerto Rico. There have been no known human melioidosis cases reported from the specific location in Arecibo where we detected it in the environment, or even that general region of the island. However, *B*. *pseudomallei* is clearly ecologically established in Puerto Rico and, as previously suggested [20], both the public and clinicians in Puerto Rico should be made more aware of it. Of note, all but one of the previous human melioidosis cases in Puerto Rico occurred in immunocompromised individuals [20]. The International Diabetes Federation stated there were over 400,600 cases of diabetes in Puerto Rico in 2017 with a total prevalence of diabetes in adults of 15.4% [84]. As diabetes is an important risk factor for melioidosis, clinicians should particularly be aware of the possibility of melioidosis in these individuals.

## Disclaimer

The findings and conclusions in this report are those of the authors and do not necessarily represent the official position of the Centers for Disease Control and Prevention.

## Supporting information

S1 Fig BEAST molecular clock estimations (chromosome 1) of eight *B*. *pseudomallei* isolates from Puerto Rico and Trinidad. BEAST phylogeny with error bars showing 95% highest posterior density (HPD); three *B*. *pseudomallei* isolates from Martinique were used as an outgroup.
(JPG)

S2 Fig Comparative genomics of *B*. *pseudomallei* from a single location (site 23). Left: Core genome phylogeny of 55 *B*. *pseudomallei* isolates obtained from site 23 containing three major clades (A-C) with a consistency index of 0.99. The phylogeny is rooted with a *B*. *thailandensis* isolate and bootstrap values are reported on nodes. Right: Presence/absence of the 58 genes that comprise the accessory genome (S5 Table) of this group of isolates; 49 of these accessory genes are in GI1, GI2, and GI3. Each cell provides the BSR values (0 represents no alignment; 1 represents an identical nucleotide alignment) for different accessory genes (columns) in the individual genomes (rows). Green text indicates *B*. *pseudomallei* isolates from soil sample 09, whereas black text represents *B*. *pseudomallei* isolates from soil sample 07. Bolded and italicized text indicates the two isolates (Bp9039 and Bp9110) included in Fig 2.
(TIF)

S1 Table Genome accession numbers, MLST, and other data for 129 *Burkholderia* spp. isolates obtained in this study. *Species identification was based upon whole genome sequence comparisons. ^a^ PS-4 previously described [6]. *B*. *ubonensis* strain resistant to meropenem (>32μg/mL). Yellow highlighted cells indicate novel MLST alleles and STs identified in this study.
(XLSX)

S2 Table Genome accession numbers for additional *Burkholderia* genomes used to construct the phylogenies presented in Figs 2–4. n/a = not applicable.
(XLSX)

S3 Table Genome accession numbers and epidemiological data for 55 *Burkholderia pseudomallei* isolates obtained in this study. All of these *B*. *pseudomallei* isolates were isolated from site 23 in the municipality of Arecibo. These 55 isolates are also presented in Fig 3 and S2 Fig.
(XLSX)

S4 Table Information on 60 sampling sites where environmental samples were collected in Puerto Rico. Soil was collected at sites 1–50 and water was collected at sites 51–60. Bolded site ID indicates the site where *B*. *pseudomallei* was isolated. * Potentially novel species; ** Soil profile downloaded for each site from USDA NRCS Web Soil Survey; n/a = not applicable.
(XLSX)

S5 Table Protein accession numbers and descriptions for the 119 *B*. *pseudomallei* accessory genes detected among 55 *Burkholderia pseudomallei* isolates obtained in this study. These genes were detected using BSR (see text). Information on the regions flanking the four genomic islands is also provided.
(XLSX)
