# Supplementary figures and images for: Burkholderia pseudomallei, the causative agent of melioidosis, is rare but ecologically established and widely dispersed in the environment in Puerto Rico
Source: PLoS Negl Trop Dis. 2019 Sep 5;13(9):e0007727. doi: 10.1371/journal.pntd.0007727 (PMC6748447; doi:10.1371/journal.pntd.0007727)

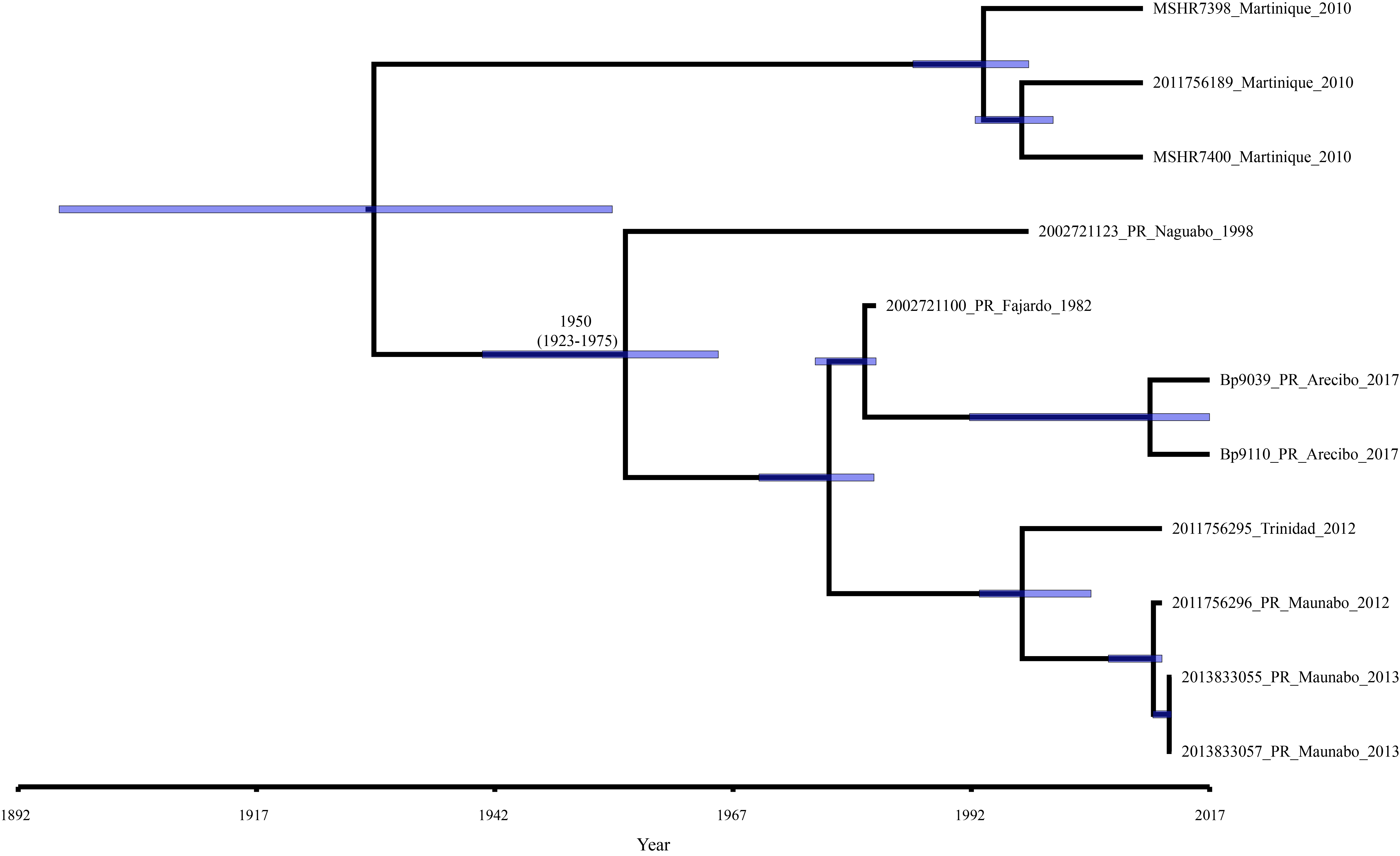

Supplement: S1 Fig — BEAST phylogeny with error bars showing 95% highest posterior density (HPD); three B. pseudomallei isolates from Martinique were used as an outgroup. (JPG) [file pntd.0007727.s001.jpg]
